# Empowering leadership as a secure base:enhancing organizational identification via age-contingent employee resilience

**DOI:** 10.3389/fpsyg.2026.1785496

**Published:** 2026-03-25

**Authors:** Yuan Zhang, Zhigang Wang, Yang Song

**Affiliations:** Business School, Zhuhai College of Science and Technology, Zhuhai, Guangdong, China

**Keywords:** age, attachment theory, employee resilience, empowering leadership, moderated mediation, organizational identification

## Abstract

**Introduction:**

In the volatile, uncertain, complex, and ambiguous (VUCA) era, employee resilience extends beyond a mere trait to serve as a critical strategic asset. Departing from the transactional logic of social exchange theory, this study adopts an attachment theory perspective to propose a paradigm shift. Within this framework, empowering leaders function as a secure base for their followers. And resilience emerges as the core mechanism through which such leadership behaviors foster a stronger sense of organizational identification. Furthermore, rather than assuming uniform effects, employee age is identified as a boundary condition that moderates the effectiveness of this process.

**Methods:**

Data were collected through a three-wave, time-lagged survey (*N* = 218) with two-week intervals among employees in the high-pressure Pearl River Delta (PRD) region of China. Hypotheses were tested using the SPSS PROCESS macro (Models 4 and 7) with 5,000 bootstrap resamples to ensure the robustness of the proposed moderated mediation framework.

**Results:**

Empowering leadership is positively associated with both employee resilience and organizational identification. Mediation analysis confirms that resilience is the key psychological mechanism linking leadership behaviors to organizational attitudes. Importantly, employee age significantly moderates the relationship between empowering leadership and resilience: the positive effect of empowering leadership on resilience is stronger for older employees and weaker for younger employees. Consequently, the indirect effect of empowering leadership on organizational identification through resilience is significantly more pronounced among older employees, supporting the hypothesized moderated mediation model.

**Discussion:**

By shifting the paradigm from a transactional “quid pro quo” framing to an attachment-based perspective, this study advances the literature on resilience by demonstrating that resilience is cultivated through relational security rather than simple reciprocity. The findings challenge the prevailing “one-size-fits-all” approach and offer an age-contingent framework for resilience management. Practical insights are provided for fostering a resilient and committed multi-generational workforce in high-intensity environments by linking micro-level leadership behaviors with macro-level organizational identification through a lifespan developmental perspective.

## 1 Introduction

The rapid evolution of modern work structures, marked by socio-technical complexity, rising job precarity, and pervasive digital transformation, has placed unprecedented psychological demands on the global workforce (Marsh et al., 2024). In this context, employee resilience, defined as the capacity to navigate adversity, recover from setbacks, and sustain proactive growth, has become a critical determinant of both individual wellbeing and organizational survival (Shin et al., 2012). This research aims to investigate how empowering leadership functions as an antecedent of organizational identification by fostering employee resilience. Moreover, the study clarifies how the strength and nature of these associations shift across the human lifespan. As organizations strive for agility in in the volatile, uncertain, complex, and ambiguous (VUCA) era, understanding the relational mechanisms that anchor employees to their firms becomes crucial.

While existing leadership literature has mainly relied on Social Exchange Theory (SET) to explain leader-follower dynamics through the lens of transactional reciprocity (Blau, 1964; Wang et al., 2023; Joo et al., 2023), the explanatory power of SET is inherently limited. SET effectively explains what is exchanged (e.g., resources, support, recognition) but remains silent on how employees internalize these exchanges during moments of adversity and distress (Cropanzano and Mitchell, 2005). It overlooks the deeper emotional foundations and Internal Working Models (IWMs) that shape how individuals interpret and respond to supportive leadership, particularly under pressure (Cooper-Thomas and Morrison, 2018).

To address this gap, this study draws on attachment theory (Bowlby, 1982; Bretherton, 1992). Attachment theory is uniquely suited to explain the cultivation of resilience because it centers on the role of a secure base (Bowlby, 1982). It is posited that a relational figure who provides psychological safety can enable exploration and recovery for individuals in the face of threat (Mikulincer and Shaver, 2015). Accordingly, empowering leaders by delegating authority and providing developmental support, fulfill the dual functions of a secure base: they encourage exploration (autonomy and growth) and offer a safe haven during distress (emotional support and protection). When leaders grant autonomy, they signal relational trust, fostering psychological safety (Frazier et al., 2017). This security allows employees to reframe challenges as opportunities for mastery rather than existential threats. This reframing process constitutes the transformative mechanism through which resilience is cultivated. Thus, while SET explains the transaction, attachment theory explains the transformation of how leadership support is internalized into a resilient self-identity that ultimately aligns with the organization (Mael and Ashforth, 1992).

The functional utility of this secure base becomes especially evident when considering the developmental stages of the modern workforce. Across the career lifespan, employee age serves as a demographic proxy for shifting developmental and relational priorities (Kooij et al., 2011). For instance, younger employees, often situated in an achievement-oriented career phase, may perceive empowerment as a normative or instrumental job feature (Aggarwal et al., 2022). In contrast, older employees, who prioritize emotional regulation and meaningful relational cues (Urry and Gross, 2010), are more likely to interpret the same empowering behaviors as profound signals of relational security. Consequently, for an older worker navigating job precarity, the leader’s trust acts as a more potent fuel for resilience than it does for their younger counterparts.

While these theoretical frameworks strongly suggest that age dictates how empowering leadership is internalized, empirical research has yet to systematically test these shifting relational dynamics as a boundary condition. Furthermore, the downstream effects of this age-contingent resilience on broader outcomes, such as organizational identification, remain unexplored. To address these empirical gaps and unpack the emotional depth of leadership, this study aims to investigate the following research questions (RQ):

*RQ1*: Does empowering leadership enhance organizational identification, and to what extent is this relationship mediated by the psychological conduit of employee resilience?

*RQ2*: Given the varying socioemotional needs across career development stages, how does employee age moderate the effectiveness of empowering leadership as a secure base for fostering resilience?

*RQ3*: Does the indirect effect of empowering leadership on organizational identification via resilience strengthen or weaken as a function of employee age, which moderates this pathway by representing differing developmental career phases?

By examining this moderated mediation model, the following three primary contributions to the field are offered:

(1) Theoretical innovation: The research advances the literature on resilience by positioning attachment theory as a robust alternative to SET, emphasizing the emotional and relational foundations through which resilience is cultivated.
(2) Mechanism elucidation: It establishes a comprehensive pathway that links micro-level behaviors, such as empowering leadership, to macro-level attitudes such as organizational identification, with resilience positioned as the transformative mechanism connecting individual support to collective identity.
(3) Boundary condition identification: The study responds to calls for age-aware human resource (HR) practices by demonstrating how lifespan developmental differences shape employees’ leadership responses, thereby offering a more nuanced understanding of “for whom” empowering leadership is most effective.

## 2 Theoretical framework and development of hypotheses

### 2.1 Attachment theory

Attachment theory posits that an inherent tendency to seek closeness to attachment figures when confronted with uncertainty or threat fundamentally guides human behavior (Bowlby, 1969). Central to this theory are the complementary concepts of the “secure base” and the “haven” (Ainsworth et al., 1978), through which attachment figures enable both adaptive exploration and emotional regulation. Specifically, a secure base provides the confidence required for individuals to explore their environment and take risks, whereas a safe haven provides reassurance, comfort, and stress alleviation in response to external challenges (Ainsworth et al., 1978).

In the modern workplace, the hierarchical nature of leader-follower relationships has been conceptualized as primary attachment relationships (Mayseless, 2010; Yip et al., 2018). Leaders are increasingly recognized as proximal attachment figures who shape employees’ IWMs (Wu and Parker, 2017). These IWMs serve as mental representations of the self as worthy of support and others as reliable (Davidovitz et al., 2007). When leaders provide consistent support and autonomy, the organization is perceived as a secure environment (Yin and Liu, 2022), thereby fostering a psychologically safe environment that enables employees to move beyond defensive postures and engage in proactive behaviors (Kahn, 1995).

### 2.2 Empowering leadership as an organizational identification driver

Empowering leadership involves sharing power, granting high levels of autonomy, and demonstrating confidence in employees’ abilities (Pearce and Sims, 2002). From the perspective of attachment theory, empowering leaders act as effective attachment figures by signaling trust and affirming employees’ sense of agency (Game and West, 2011). They create a secure base that promotes employees’ professional exploration and fosters deeper psychological investment in their work (Game and West, 2011).

This leadership style serves as a primary driver of employees’ organizational identification (Burhan and Khan, 2024). Although empowering practices are traditionally recognized for their ability to enhance functional work outcomes (e.g., Lorinkova et al., 2013; Wen et al., 2023), their effects are understood to extend beyond task performance. Organizational identification is defined as a psychological state of oneness in which employees’ self-concepts are shaped through their organizational membership (Mael and Ashforth, 1992). Because leaders are often perceived as representatives of the organization, the support and relational security provided by an empowering leader are frequently generalized to the organization as a whole (Eisenberger et al., 2001).

Kim and Beehr (2021) provide empirical support for this argument, showing that the autonomy and developmental support inherent in empowering leadership elicit positive affective responses and foster deep psychological commitment. From an attachment perspective, these resources are understood not only as functional tools but also as tangible expressions of a secure base, where autonomy signals a “safe-to-fail” environment and developmental support functions as a safety net (Richards and Schat, 2011). This relational security reduces self-protective barriers and encourages employees to integrate the organization into their self-concept, as reflected in the affective outcomes reported by Kim and Beehr (2021). In a study within the Spanish military, Navas-Jiménez et al. (2024) found that secure-base leadership fosters a greater alignment between personal and collective values, thereby boosting organizational identification.

Complementing this attachment-based account, SET further suggests that empowering leadership strengthens organizational identification by cultivating high-quality exchange relationships (Blau, 1964; Cropanzano and Mitchell, 2005). By granting autonomy, expressing trust, and offering support, empowering leaders initiate a cycle of positive reciprocity. Because leaders are viewed as agents of the organization, such behaviors signal organizational investment in employees (Eisenberger et al., 2001). In response, employees develop a sense of reciprocal obligation and strengthen positive attitudinal outcomes, including a stronger psychological attachment to the organization (Rhoades and Eisenberger, 2002). As a result, employees are more prone to incorporate organizational membership into their self-concept, leading to higher levels of organizational identification. Based on these findings, it is posited that:

*H1*: Empowering leadership is associated with higher levels of organizational identification.

### 2.3 Empowering leadership in fostering employee resilience

Rather than viewing employee resilience as a static characteristic, current scholarship conceptualizes it as an evolving development path. It is characterized by an individual’s ability to managing psychological assets for adapting and thriving amidst professional challenges (Shin et al., 2012). Empowering leadership is proposed to serve as a key antecedent of this capacity by creating a secure base environment that supports both proactive exploration and psychological buffering, thereby enabling employees to navigate challenges and enhance their adaptive potential.

From an attachment perspective, empowering leaders fosters employee resilience through two complementary mechanisms: functional exploration and resource preservation (Bowlby, 1969). By granting autonomy and promoting self-directed decision-making, leaders provide the security necessary for employees to engage in exploration, creating a secure base that enables experimentation with novel solutions and the expansion of proactive problem-solving skills (Wu and Parker, 2017). In this process, internal resources such as self-efficacy and personal agency are strengthened. As noted by Kim and Beehr (2023), a sense of mastery is cultivated when leadership validates an employee’s discretionary power, which is essential for effectively navigating complex workplace stressors.

Second, empowering leadership acts as a safety net that buffers the depletion of psychological resources during failure (Wu and Parker, 2017; Kim and Beehr, 2021). In an empowering climate, errors are reframed as learning opportunities rather than triggers for punitive measures (Yin and Liu, 2022). This shift ensures that adversity prompts a sustained psychological rebound instead of triggering self-protective withdrawal (Yin and Liu, 2022). This function is particularly critical in high-stakes environments. For example, Khan et al. (2025) showed that the relational support inherent in empowering leadership prevents emotional exhaustion and helps maintain resilience in emergency service organizations, where adversity is constant.

Furthermore, empowering leaders’ secure base influences the cognitive processing of stress. Navas-Jiménez et al. (2024) argue that such leadership facilitates the adaptive reframing of stress. By ensuring that support is readily accessible, empowering leaders transform perceived threats into opportunities for mastery (Yin and Liu, 2022). This shift in perception not only reduces intensity induced by stress but also enhances followers’ perceived competence in overcoming workplace difficulties (Yin and Liu, 2022).

Accordingly, empowering leadership bolsters employees’ capacity for psychological rebound amidst adversity. This recovery is driven by a synergy between external relational anchors and internal confidence. Such leaders establish a secure base that encourages autonomous exploration while buffering the risks of failure, thereby fostering the foundational psychological systems essential for resilience (Wu and Parker, 2017). Extending this reasoning, SET offers a complementary lens by emphasizing the reciprocal dynamics embedded in such supportive relationships (Blau, 1964). Empowering behaviors signal organizational care and investment, which strengthens perceived socio-emotional support and fosters felt obligation (Cropanzano and Mitchell, 2005; Eisenberger et al., 2001). Consequently, employees tend to reciprocate with sustained effort, adaptive persistence, and constructive coping when facing adversity (Arshad et al., 2022), which in turn promotes recovery from setbacks and resilience development. Thus, it is posited that:

*H2*: Empowering leadership is associated with higher employee resilience levels.

### 2.4 Employee resilience and organizational identification

Beyond its functional role in coping with adversity, employee resilience is proposed to serve as a critical psychological bridge to organizational identification through internalization. From the perspective of attachment theory, interactions between the individual and a secure base, represented by the empowering leader, shape the individual’s IWM—the cognitive representations of the self and the social environment (Bowlby, 1982).

When employees effectively leverage the resources provided by an empowering leader to recover from setbacks, their resilience is not perceived in isolation. Rather, growth is understood as a co-constructed outcome of the employees’ own agency and the organization’s support (Yin and Liu, 2022). Drawing on the internalization mechanism within attachment frameworks, as the organization is experienced as a consistent and reliable source of security that sustains resilience, employees progress beyond mere gratitude toward the integration of organizational membership into their self-concept.

Specifically, employees identify the organization as a secure base that actively facilitates the development of their “resilient self.” This perception drives individuals to integrate organizational identity into their self-concept to maintain long-term psychological stability. This perspective provides a nuanced alternative to existing research, such as Mao et al. (2022), which indicates that resilience precedes identification. In the context of empowerment, the lived experience of being resilient within a supportive collective serves as a validating mechanism. This process strengthens the bond between the individual and the organization, ultimately leading employees to perceive the organization’s values and outcomes as closely aligned with their own (Mael and Ashforth, 1992).

SET provides an additional explanation for why resilience may translate into stronger organizational identification (Blau, 1964; Cropanzano and Mitchell, 2005). When employees successfully recover from adversity, they are likely to attribute part of this adaptive capacity to the support and resources provided by the organization (Eisenberger et al., 1986; Rhoades and Eisenberger, 2002). Such perceived investment fosters reciprocal obligation and motivates employees to respond with deeper attitudinal attachment rather than merely task performance (Gouldner, 1960). Over time, this reciprocal dynamic enhances the relational bond between employees and the organization, making organizational membership to the self-concept and thereby increasing organization identification (Mael and Ashforth, 1992). Based on this reasoning, we posited that:

*H3*: Employee resilience is associated with higher organizational identification levels.

### 2.5 Mediating role of resilience

A mediation model is proposed in which employee resilience functions as the key psychological conduit linking empowering leadership behaviors to organizational attitudes. While empowering leadership supplies the external structural and relational conditions, serving as a “secure base,” the internal development of resilience ultimately anchors the employee’s identity to the organization.

From an attachment perspective, this mediation process represents the internalization of relational resources. The empowering leader initiates the cycle by providing a secure base that employees internalize as a lasting capacity to manage stress and pursue novel professional challenges (Yin and Liu, 2022). Once internalized, resilience fosters a profound sense of both affective and cognitive alignment with the organization.

Instead of exerting a simple direct effect, empowerment leadership is proposed to influence organizational identification through the mediating role of resilience. An identity shift occurs when employees perceive their capacity to recover and thrive as a co-constructed outcome of their own agency and the support provided by the leader, leading them to view the organization as an integral component of their resilient self. In this way, resilience functions as the transformative mechanism that converts external managerial support into a lasting internal bond with the collective.

Complementing the attachment-based explanation, SET (Blau, 1964; Cropanzano and Mitchell, 2005) provides an additional rationale for the mediating role of resilience. Empowering leadership signals organizational investment through autonomy, trust and developmental support (Zhang and Bartol, 2010), thereby fostering high-quality exchange relationships (Cropanzano and Mitchell, 2005). Because leaders are viewed as agents of the organization, such behaviors enhance employees’ perceptions of organizational support (Eisenberger et al., 1986; Rhoades and Eisenberger, 2002). Within supervisor-subordinate relationships, employees experience socio-emotional resources that enhance their confidence and adaptive capacity when confronting adversity. When employees effectively mobilize psychological resources to maintain performance under pressure, they engage in a reciprocal process. This dynamic transforms empowering leadership behaviors into a lasting psychological bond, with resilience acting as the bridge that converts transactional support into durable psychological attachment (Gouldner, 1960). Rather than operating as a simple direct effect, empowering leadership thus influences identification through the development of resilience embedded in reciprocal social exchange. Therefore, it is hypothesized that:

*H4*: Employee resilience mediates the relationship between empowering leadership and organizational identification.

### 2.6 The moderating role of employee age

Beyond its direct and mediating effects, the strength of the relationship between empowering leadership and employee resilience is contingent on employee age. From the perspective of attachment theory, age is viewed not merely as a chronological measure but also as an indicator of the maturation and refinement of IWMs, the cognitive-affective representations of the self and others that shape the responses of individuals to social support (Bowlby, 1969; Shaver and Mikulincer, 2007).

According to SST, as individuals perceive their remaining time as increasingly limited with age, priorities shift from instrumental, knowledge-focused goals toward emotionally meaningful objectives and supportive networks (Carstensen et al., 1999). Therefore, for older employees, the leader extends beyond the role of task supervisor to function as a vital secure base that offers emotional validation.

Empowering leadership, marked by the delegation of authority and the expression of confidence, closely aligns with the increased need for inclusion and respect observed in the late-career stages of the profession. When autonomy is granted to older employees, it is perceived as a meaningful relational investment, reinforcing their secure attachment to the leader and creating a strong psychological buffer that substantially enhances their resilience against workplace adversity (Urry and Gross, 2010).

In contrast, younger employees often possess IWMs that are more egalitarian and self-reliant compared to older employees. Younger employees, especially in more modern, low power distance work cultures, often view autonomy and empowerment as inherent aspects of their job roles. This perception aligns with studies showing that younger employees, particularly those from younger generations (e.g., Millennials and Generation Z), have a preference for flat organizational structures and autonomy in decision-making (Ng et al., 2010; Twenge et al., 2012). Younger employees tend to perceive empowerment as a standard job expectation rather than a distinctive relational investment (Gao, 2023). For this demographic, self-expression and personal growth take precedence over vertical power dynamics (Gao, 2023). Empowerment is therefore perceived more as an instrumental resource for achieving goals than as a medium for emotional connection with leaders (Gao, 2023). This reduced emotional intensity of the leadership empowerment dynamic is predicted to contribute to a less profound effect of resilience for younger employees.

Moreover, generational differences in IWMs further shape how empowerment is perceived. In traditional high power distance cultures like China, older employees often have hierarchical relational schemas (Yang, 2004). For these employees, empowering leadership constitutes a positive expectation violation, as the unexpected shift from directive control to empowerment serves as a salient attachment cue, signaling that the leader regards them as a valued partner. This high-quality relational bond provides the emotional resources necessary to sustain psychological rebound. This effect is less pronounced for younger employees, who are more likely to view empowerment as a standard job expectation rather than a relational investment (Gao, 2023). Based on this reasoning, we posit that:

*H5*: Employee age moderates the relationship between empowering leadership and resilience, with older employees having a stronger positive effect.

### 2.7 Moderated mediation model

The proposed age-contingent effect on the relationship between leadership and resilience indicates a more complex, moderated mediation pattern. Resilience functions as the primary mechanism translating empowering leadership into organizational identification. Accordingly, any boundary condition affecting the initial link between leadership and resilience will ultimately alter the magnitude of the entire indirect effect.

Based on attachment theory, the leader functions as proximal attachment figures who provide secure-base and safe-haven functions, especially under conditions of threat, uncertainty, or heightened dependency (Mayseless, 2010; Game and West, 2011). When empowering leaders delegate authority, express confidence, and offer developmental support, employees perceive more than mere managerial routines. Instead, they interpret these behaviors as attachment-relevant cues, signaling the leader’s reliability and availability. This interpretation facilitates the internalization of security. Employees become more resilient as they are more willing to explore, recover from setbacks, and maintain adaptive functioning. Over time, this resilience is more likely to be attributed to, and embedded with, the organizational context represented by the leader, therefore strengthening organizational identification through an empowering leadership → resilience → organizational identification linkage.

Crucially, the present model specifies employee age as a boundary condition because age systematically shifts the meaning employees assign to relational cues from leaders. As the future time horizon becomes more constrained with age, SST suggests a fundamental realignment toward emotionally significant objectives (Carstensen et al., 1999). This realignment makes high-quality interpersonal connections and security-based cues more relevant within the individual’s cognitive framework (Carstensen et al., 1999). In this sense, empowering leadership should be more likely to be processed as a secure-base signal among older employees, who are more attuned to trust, respect, and emotional validation in workplace relationships.

In contrast, younger employees, particularly those socialized into flatter, autonomy-emphasizing norms, may treat empowerment as a standard job feature and interpret leaders’ grant of autonomy primarily in functional or instrumental terms (Ng et al., 2010). As a result, the same empowering behaviors may generate weaker attachment-related internalization and thus lower degree of resilience for younger employees.

Since resilience translates empowering leadership into organizational identification, the age-based differences at the start of this process are pivotal. These differences ensure that the total indirect effect is not uniform but rather fluctuates according to the employee’s age. Specifically, when empowering leadership is interpreted as a stronger secure-base cue among older employees, resilience becomes a more powerful resource. It helps employees connect themselves to the organization, producing a stronger empowering leadership → resilience → organizational identification pathway. Conversely, when empowerment is interpreted as routine professional practice among younger employees, the resilience gain is smaller, weakening the transmission into organizational identification. Accordingly, it is hypothesized that:

*H6*: Employee age moderates the indirect effect of empowering leadership on organizational identification through resilience, with the effect being stronger for older employees.

**Figure 1:**
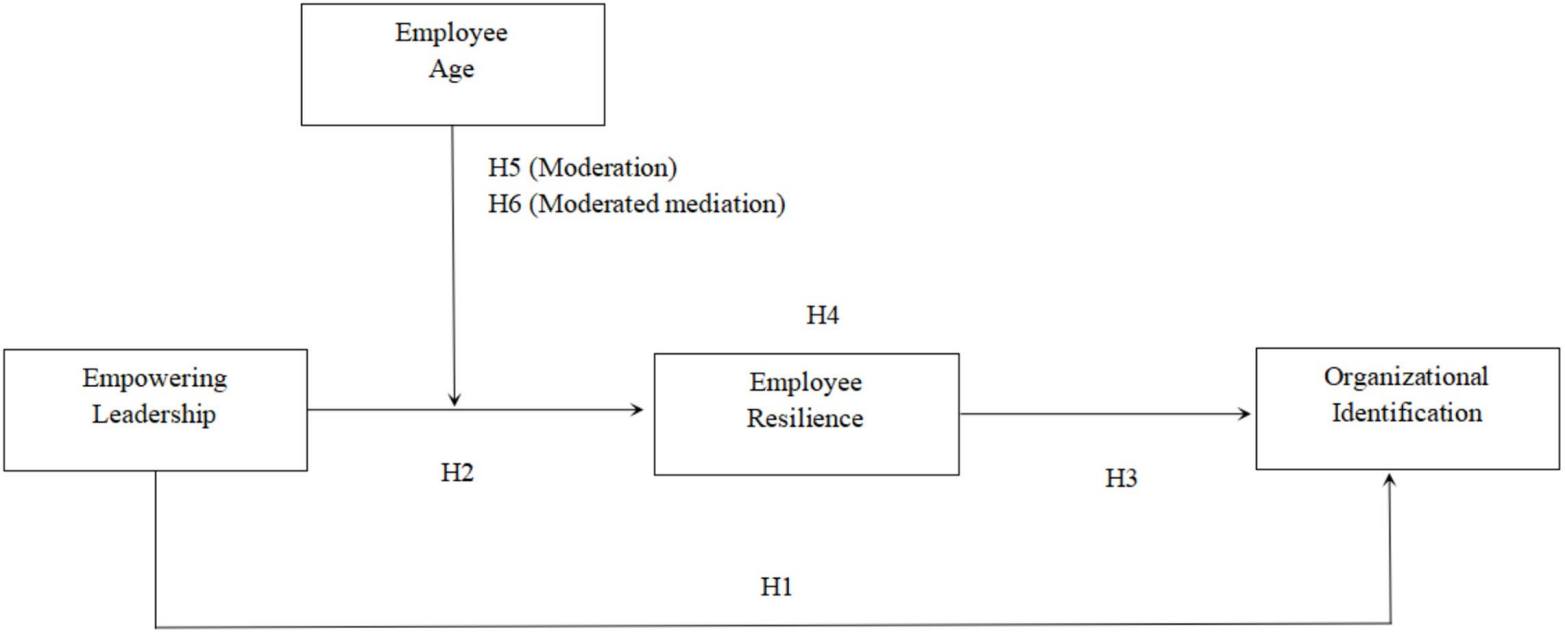
Theoretical model.

Figure 1 depicts the theoretical framework.

## 3 Methods

### 3.1 Data collection and sample characteristics

Data collection was performed in the Pearl River Delta (PRD) of Southern China, a region comprising nine major industrial cities, such as Guangzhou, Shenzhen, and Dongguan, which is recognized as one of the world’s most densely urbanized and economically dynamic clusters. Historically known as the “World’s Factory,” the PRD has rapidly transformed into a global hub for high-technology manufacturing, financial services, and digital innovation. This intense economic environment has cultivated a distinctive high-pressure, high-velocity organizational climate in which employees routinely navigate rapid market shifts and frequent organizational restructuring. Such conditions make the PRD an ideal socio-economic context for examining the mechanisms underlying employee resilience and the effectiveness of empowering leadership.

This study was conducted in full compliance with the ethical standards outlined in the 1964 Declaration of Helsinki and its subsequent amendments. The research protocol was reviewed and approved by the Institutional Research Ethics Committee of the Business School, Zhuhai College of Science and Technology, before data collection. The following ethical safeguards were implemented:

(1) Voluntary participation and informed consent: Participation was entirely voluntary, and all participants received a plain language statement and provided informed consent before Time 1.
(2) Non-invasive nature: The study was purely psychological and survey-based, with no invasive medical procedures, physiological testing, or physical experiments involving human participants.
(3) Anonymity and data protection: Participants created unique identification codes to ensure anonymity while enabling longitudinal tracking. To ensure confidentiality, the data were maintained within a repository protected with a password and accessible solely by the authorized research team.

To reduce Common Method Bias (CMB) and establish temporal precedence, a three-wave, time-lagged survey design was employed, with two-week intervals between each wave, following the procedural recommendations of Podsakoff et al. (2003). A two-week interval was specifically chosen as an optimal time lag to balance methodological and theoretical considerations. Methodologically, it is sufficiently long to allow temporary mood states and memory anchors from the previous survey to dissipate, yet short enough to ensure the overarching organizational context and leader-follower relationship remain stable (Dormann and Griffin, 2015). The time-lagged design is also highly consistent with recent empirical designs examining leadership and employee psychological outcomes (e.g., Abedini et al., 2024; Fang et al., 2025). Theoretically, this temporal separation reflects the psychological processes posited by attachment theory. At Time 1 (T1), empowering leaders establish a secure base for employees. However, the mobilization of psychological resources required to build resilience unfolds over time, manifesting at Time 2 (T2). Subsequently, the internalization of this resilience into a stable sense of organizational identification occurs gradually as employees reflect on their supportive work environment, captured at Time 3 (T3). The data collection procedure was conducted as follows:

(1) T1: Participants were asked to assess their supervisors’ empowering leadership behaviors and provide demographic information (e.g., age, gender, and organizational tenure). Each participant created a unique identification code to facilitate matching across survey waves while preserving anonymity.
(2) T2: Two weeks later, the same participants were contacted to report their employee resilience levels.
(3) T3: After an additional two-week interval, participants self-reported their organizational identification levels.

To obtain a heterogeneous sample reflective of the broader working population, participants were recruited through a combination of the research team’s professional networks and referrals from corporate partners. Invitations were distributed to employees across various industries (e.g., manufacturing, technology and services) in the PRD region to enhance the generalizability of the findings. A total of 243 employees initially agreed to participate in this study.

Each participant was assigned a distinct identification code to facilitate confidential tracking. After filtering out individuals who failed to participate in all three survey waves, 218 valid responses were retained, yielding an effective response rate of 89.70% and indicating low attrition for a time-lagged study. The demographic characteristics of the sample are presented in Table 1.

**Table 1:** Sample demographic characteristics.

Characteristic	Category	Frequency (*N*)	Percentage (%)
Gender	Male	84	38.54
	Female	134	61.46
Age	22–30 years	126	57.80
	31–40 years	76	34.86
	41–52 years	16	7.34
Marital status	Married	136	62.39
	Single	82	37.61
Education level	College degree	37	16.97
	Bachelor degree	169	77.52
	Master’s degree or above	12	5.51
Organizational tenure	Less than 1 year	11	5.05
	1–5 years	175	80.28
	6–10 years	22	10.09
	11–21 years	10	4.58
Industry	Manufacturing	86	39.45
	Internet and technology	80	36.70
	Service	52	23.85

*N* = 218.

The final sample represented a cross-section of industries characteristic of the PRD’s economic landscape. Manufacturing comprised the largest segment at 39.45% (*n* = 86), reflecting the region’s deep industrial heritage as the historic “World Factory.” Technology and internet services accounted for 36.70% (*n* = 80), capturing the region’s ongoing transformation into a global hub for digital innovation and high-tech development. The service sector constituted 23.85% (*n* = 52), primarily comprising financial services, with the remaining distributed across other sectors. This industry composition accurately captures the PRD’s dual identity as both an established manufacturing powerhouse and emerging innovation-driven economy. The substantial representation of both traditional manufacturing and modern technology sectors enhances the generalizability of our findings across the region’s dominant industries and reflects the broader economic transition occuring in one of China’s most dynamic industrial zones.

### 3.2 Measurement

All constructs in this study were measured using established scales that have been widely validated in the literature on organizational behavior. To ensure both conceptual equivalence and linguistic accuracy in the Chinese context, the translation and back-translation procedure recommended by Brislin (1970) was followed. The scales were first translated into Chinese by a bilingual researcher and then back-translated into English by an independent researcher to verify consistency. Unless otherwise indicated, all items were rated on a 5-point Likert scale, ranging from 1 (strongly disagree) to 5 (strongly agree).

#### 3.2.1 Empowering leadership (T1)

Empowering leadership was measured at T1 using the 22-item scale developed by Pearce and Sims (2002), which captures six key dimensions: (1) encouraging self-development, (2) encouraging self-reward, (3) encouraging independent action, (4) encouraging self-expectation, (5) encouraging self-goal setting, and (6) encouraging opportunity thinking. The overall scale demonstrated high reliability, with a Cronbach’s alpha of 0.92, and all sub-dimensions exceeded the 0.70 threshold. A sample item is: “My supervisor encourages me to find solutions to my own problems.”

#### 3.2.2 Employee resilience (T2)

Employee resilience was measured at T2 using the 4-item resilience subscale from the Psychological Capital Questionnaire developed by Luthans et al. (2007), which assesses an individual’s capacity to recover from adversity and maintain growth. The scale demonstrated good internal consistency, with a Cronbach’s alpha of 0.80. A sample item is: “I usually take stressful things at work in stride.”

#### 3.2.3 Organizational identification (T3)

Organizational identification was assessed at T3 using the 6-item scale developed by Mael and Ashforth (1992), which evaluates the degree to which individuals define themselves through their organizational membership. In this study, the scale demonstrated acceptable reliability, with a Cronbach’s alpha of 0.75. A sample item is: “When someone criticizes my organization, it feels like a personal insult.”

#### 3.2.4 Employee age (moderator, T1)

Age was treated as a continuous variable in accordance with SST, with participants reporting their chronological age in years (T1). This continuous approach enables a more precise estimation of developmental shifts in socioemotional goals than categorical age groups.

#### 3.2.5 Control variables (T1)

To ensure the robustness of the findings and account for potential confounding factors, several demographic variables (T1) were included as controls based on previous research in organizational psychology:

(1) Organizational tenure: measured as the number of years an employee has been with the firm.
(2) Gender: coded as 1 = male and 2 = female.
(3) Marital status: coded as 1 = married and 2 = single; and
(4) Educational background: coded as 1 = college degree, 2 = bachelor’s degree, and 3 = master’s degree or higher.

### 3.3 Statistical analysis strategy

The PROCESS macro for SPSS (Version 4.1; Hayes, 2018) was employed to test the hypothesized moderated mediation model. Although structural equation modeling (SEM) is commonly used to account for measurement error through latent constructs, path analysis via PROCESS was considered more suitable for several reasons. First, given the sample size of 218, ordinary least square based path analysis in PROCESS provides greater statistical power and stability than SEM. SEM typically requires significantly larger sample to achieve reliable convergence and stable fit indices, especially when testing complex interaction effects which often lead to estimation problems or improper solutions in a latent variable framework (Kline, 2023).

Second, PROCESS is specifically optimized for testing conditional indirect effects. It offers a more parsimonious approach by using observed composite scores, which avoids the computational complexity of latent interaction terms while providing specialized outputs like the Index of Moderated Mediation (Hayes, 2018). This ensures a more direct and robust interpretation of the psychological mechanisms.

Finally, to account for potential non-normality, a non-parametric bootstrapping procedure with 5,000 resamples was employed to generate 95% bias-corrected confidence intervals (CIs). This method yields more rigorous results for mediation and moderation than traditional approaches (Preacher and Hayes, 2008).

## 4 Results

### 4.1 Methodological validation

#### 4.1.1 Common method bias

To ensure the integrity of the findings, a series of methodological checks were conducted to assess and mitigate the potential impact of CMB. Both procedural and statistical approaches were employed to address this concern (Podsakoff et al., 2012).

From a procedural standpoint, several measures were implemented during the research design phase to minimize CMB. First, temporal separation was introduced by collecting data in three waves with two-week intervals between each wave. This design reduced the likelihood of consistency motifs and recall biases, as it helped diminish the impact of memory effects over time (Podsakoff et al., 2012). Second, respondent anonymity was guaranteed. The instructions explicitly stated that no response could be considered right or wrong, which encouraged honest and unbiased responses. While reverse-coded items were not included in the final scales due to the original instrument design, the combination of temporal separation and anonymity serves as effective procedural remedies to minimize CMB (Podsakoff et al., 2012).

In terms of statistical diagnostics, several analyses were conducted to assess the extent of CMB. First, Harman’s one-factor test was executed by loading all study constructs into a principal component analysis without rotation. The results indicated that the first extracted factor accounted for 34.04% of the total variance, well below the 40% threshold recommended by Podsakoff et al. (2003), indicating that CMB was not a significant concern in this study.

However, it is important to note that Harman’s test, while useful for an initial assessment, has its limitations in detecting all forms of CMB, particularly when multiple latent variables are involved (Baumgartner et al., 2021). Therefore, as a more rigorous approach, we compared the hypothesized measurement model with a one-factor model, in which all items were loaded onto a single latent factor (Table 2 in Section 4.1.2). This one-factor model exhibited significantly poorer fit compared to the baseline three-factor model: the chi-square difference relative to degrees of freedom was substantial (Δ*χ*^2^/Δdf = 27.97). Moreover, its absolute fit indices fell well below acceptable thresholds (Hu and Bentler, 1999). This pronounced degradation in model fit indicates that a single method factor does not account for the majority of the covariance among the measures (Podsakoff et al., 2003). It provides further evidence that CMB does not present a pervasive issue in this dataset (Podsakoff et al., 2003).

**Table 2:** Comparison of measurement models.

Model	Description	*χ* ^2^	df	*χ*^2^/df	Δ*χ*^2^/Δdf	CFI	TLI	RMSEA	SRMR
Model 1	Three-factor model (EL, Res, OI)	614.68	455	1.35	-	0.93	0.93	0.04	0.05
Model 2	Two-factor model (EL + Res, OI)	761.57	463	1.64	18.36	0.87	0.86	0.05	0.06
Model 3	Two-factor model (EL, Res + OI)	685.70	457	1.50	35.51	0.90	0.89	0.05	0.06
Model 4	One-factor model (All combined)	866.42	464	1.87	27.97	0.83	0.82	0.06	0.06

N, 218; χ^2^, Chi-square, df, degree of freedom, Δ*χ*^2^/Δdf is the ratio of the differences in *χ*^2^ to the difference in df, compared with the baseline Model 1. EL, empowering leadership, Res, resilience, OI, organizational identification. CFI, Comparative Fit Index, TLI, Tucker-Lewis Index, RMSEA, Root Mean Square Error of Approximation, SRMR, Standardized Root Mean Square Residual.

Additionally, an examination of the correlation matrix revealed a pattern consistent with the absence of severe CMB. If CMB were a serious concern, we would expect to see uniformly high correlations among all variables. However, the correlations among the key variables ranged from 0.54 to 0.70 (Table 3 in Section 4.2), which are moderate and align with theoretical expectations. Moreover, the correlations between these variables and demographic characteristics (e.g., gender, tenure) were predominantly low and non-significant (e.g., empowering leadership with age: *r* = 0.05, n.s.). This pattern, with many coefficients below 0.30, further supports that CMB is unlikely to systematically inflate the observed relationships (Podsakoff et al., 2003).

**Table 3:** Means, standard deviations, and correlations among variables.

Variables	Mean	SD	1	2	3	4	5	6	7	8
1. Gender	1.61	0.49	–							
2. Marriage status	1.38	0.49	0.01	–						
3. Educational background	1.89	0.46	0.05	0.07	–					
4. Organizational tenure	3.68	3.68	−0.03	−0.34^**^	−0.24^**^	–				
5. EL	3.87	0.50	−0.07	−0.16^*^	−0.12	0.10	(0.92)			
6. Resilience	3.91	0.60	−0.06	−0.03	0.00	−0.01	0.58^**^	(0.80)		
7. OI	3.90	0.53	−0.02	−0.18^**^	−0.04	0.07	0.70^**^	0.54^**^	(0.75)	
8. Age	30.64	6.18	−0.11	−0.45^**^	−0.29^**^	0.65^**^	0.05	−0.09	0.02	–

*N* = 218. ^*^p < 0.05, ^**^p < 0.01 (two-tailed). SD, standard deviation; Gender: 1. male, 2. female; Marital status: 1. married, 2. single; Education: 1. college degree, 2. bachelor’s degree, 3. master’s degree or above; EL, empowering leadership, OI, organizational identification. Cronbach’s *α* is shown in parentheses on the diagonal.

Thus, the combination of procedural controls and multiple statistical diagnostics and multiple statistical diagnostics all suggest that CMB does not pose a significant threat to the validity of the findings.

#### 4.1.2 Confirmatory factor analysis

Confirmatory factor analysis was conducted using Mplus 7.0 to assess the distinctiveness of the study constructs. The hypothesized three-factor model demonstrated an excellent fit to the data, with *χ*^2^ = 614.68, *χ*^2^/df = 1.35 (below the 3.0 threshold), RMSEA = 0.04 (below 0.08), CFI = 0.93, and TLI = 0.93 (both above 0.90), with SRMR = 0.05 (below 0.08), collectively indicating a strong model fit according to Hu and Bentler’s (1999) criteria. Alternative models, such as those combining factors, showed significantly poorer fit, thereby confirming the discriminant validity of the constructs (Table 2).

#### 4.1.3 Sample adequacy and statistical power

To evaluate the statistical adequacy of the sample size (*N* = 218), a post-hoc power analysis was conducted using G^*^Power 3.1.9.7 (Faul et al., 2009).

In the first stage (outcome: resilience), in which the interaction between empowering leadership and age was examined, the overall model explained 37% of the variance (*R*^2^ = 0.37), corresponding to a large effect size (*f*^2^ = 0.59). With seven predictors included, the achieved statistical power (1 − *β*) exceeded 0.99, which is well above the conventional threshold of 0.80 (Cohen, 1988, 1992). For the interaction term (empowering leadership×age), which accounted for an incremental *R*^2^ of 0.02 (*f*^2^ = 0.03), the achieved power was 0.76. Given that detecting interaction effects in field research is typically difficult due to range restriction and measurement error (McClelland and Judd, 1993; Aguinis et al., 2005), this level of power indicates adequate sensitivity to test the proposed moderation hypothesis (H5).

In the second stage (outcome: organizational identification), the model explained 53% of the variance (*R*^2^ = 0.53), which corresponds to a large effect size (*f*^2^ = 1.13). With six predictors included, the achieved statistical power (1-*β*) exceeded 0.99. Taken together, these results indicate that the sample size of 218 provided sufficient statistical power to reliably detect the hypothesized mediation and moderation effects.

### 4.2 Preliminary analysis

Table 3 shows the means, standard deviations, and Pearson correlation coefficients for all study variables. Participants were 30.64 years old on average and had an organizational tenure of 3.68 years. The mean values for the focal constructs, empowering leadership (Mean = 3.87), employee resilience (Mean = 3.91), and organizational identification (Mean = 3.90), all exceeded the scale midpoint, indicating relatively high levels of perceived empowering leadership, psychological resilience, and organizational identification among employees in the PRD region.

The correlation analysis offered preliminary evidence that was consistent with the following theoretical framework:

(1) Leadership and key outcomes: As anticipated, empowering leadership exhibited significant positive correlations with employee resilience (*r* = 0.58, *p* < 0.01) and organizational identification (r = 0.70, *p* < 0.01), thereby providing preliminary support for H1 and H2.
(2) Resilience and identification: Employee resilience was positively and significantly associated with organizational identification (*r* = 0.54, *p* < 0.01), thereby supporting H3.
(3) Mediation path prerequisites: The significant inter-correlations among empowering leadership, employee resilience, and organizational identification meet the mediation analysis prerequisites (Baron and Kenny, 1986), thereby paving the way for the formal testing of H4 concerning the mediating role of resilience.

Regarding the moderator, employee age, the analysis showed a strong positive correlation with organizational tenure (*r* = 0.65, *p* < 0.01), consistent with established career development patterns. Age was not significantly correlated with empowering leadership (*r* = 0.05, *p* > 0.05) or employee resilience (*r* = −0.09, *p* > 0.05). Such non-significant zero-order associations are often desirable in moderated mediation models because they indicate that the moderator operates as a boundary condition that shapes the strength of relationships (i.e., slope differences) rather than directly influencing the levels of the focal variables. This pattern provides a robust statistical basis for testing the hypotheses of moderation and moderated mediation (H5 and H6).

### 4.3 Hypothesis testing

#### 4.3.1 Analysis of direct and mediating effects

To test the direct and mediation hypotheses (H1-H4), Model 4 of the PROCESS macro (Hayes, 2018) was employed. After controlling for demographic variables, the results (Table 4) indicated that: (1) empowering leadership significantly and positively predicted organizational identification (*β* = 0.70, *p* < 0.001), supporting H1; (2) empowering leadership had a significant positive effect on employee resilience (*β* = 0.60, *p* < 0.001), supporting H2; and (3) employee resilience was positively associated with organizational identification (*β* = 0.21, *p* < 0.001), supporting H3.

**Table 4:** Regression results for the direct and mediation models.

Variable	Model 1: resilience		Model 2: organizational identification (direct effect)		Model 3: organizational identification (full model)	
	*β*	*t*-value	*β*	*t*-value	*β*	*t*-value
Predictors						
Gender	−0.02	−0.44	0.02	0.46	0.03	0.58
Marital status	0.04	0.73	−0.08	−1.58	−0.09	−1.80
Education	0.06	1.05	0.05	0.91	0.03	0.67
Tenure	−0.05	−0.75	−0.15	−0.28	−0.01	−0.11
Empowering leadership	0.60	10.49^***^	0.70	14.13^***^	0.58	9.70^***^
Resilience					0.21	3.56^***^

Model summary			
*R*	0.59	0.71	0.73
*R* ^2^	0.34	0.50	0.53
*F*-value	22.33	42.97	39.90

*N* = 218. ^***^p < 0.001 (two-tailed).

The indirect effect of empowering leadership on organizational identification via employee resilience was examined using 5,000 bootstrap resamples. The indirect effect was statistically significant (Effect = 0.13, Boot SE = 0.04, 95% CI [0.06, 0.22]), as the 95% bias-corrected CI did not include zero (Table 5). The indirect effect accounted for approximately 17.57% of the total effect, indicating that employee resilience partially mediates the relationship between empowering leadership and organizational identification, thereby providing full support for H4.

**Table 5:** Total, direct and indirect effects of empowering leadership on organizational identification.

Effect type	Effect size	Boot SE	Boot LLCI	Boot ULCI
Total effect	0.74	0.05	0.64	0.84
Direct effect	0.61	0.06	0.49	0.73
Indirect effect	0.13	0.04	0.06	0.22

Bootstrap samples = 5,000. Confidence intervals are bias-corrected 95% intervals. Boot SE, Bootstrap Standard Error; LLCI, Lower Limit of Confidence Interval; ULCI, Upper Limit of Confidence Interval.

#### 4.3.2 Testing the moderated mediation effect

Model 7 of the PROCESS macro (Hayes, 2018) was used to examine the moderating role of employee age (H5) and the full moderated mediation model (H6). Unstandardized coefficients (*b*) were reported in accordance with recommendations for models involving interaction terms (Hayes, 2018).

First, we examined the interaction between empowering leadership and employee age on resilience. The results revealed a significant interaction effect (Table 6, *b* = 0.03, *p* < 0.01), supporting H5. This suggests that age moderates the relationship between empowering leadership and employee resilience. To evaluate the practical magnitude of this moderation, we examined the proportion of variance explained by the interaction term. The interaction accounted for a significant incremental increase in the explained variance of resilience (Δ*R*^2^ = 0.02, *p* < 0.05). It indicates that the interaction uniquely accounts for 2% of the variance in resilience beyond the main effects. This incremental variance, while modest in absolute terms, is consistent with typical effect sizes for moderation effects in field studies (Aguinis et al., 2005) and represents a practically meaningful boundary condition.

**Table 6:** Regression results for the moderated mediation model.

Variable	Model 1: resilience (mediator)		Model 2: organizational identification (outcome)	
	*b*	*t*	*b*	*t*
Predictors				
Gender	−0.02	−0.32	0.03	0.58
Marital status	0.06	0.81	−0.10	−1.80
Education	0.67	0.88	0.04	0.67
Tenure	−0.00	−0.26	−0.00	−0.11
Empowering leadership	0.72	10.64^***^	0.61	9.70^***^
Resilience				
Age	−0.01	−0.64		
Empowering Leadership × Age	0.03	2.49^**^		

Model summary		
R	0.61	0.73
*R* ^2^	0.37	0.53
*F*-value	17.62	39.90

	*b*	Boot SE	LLCI	ULCI
Conditional effect analysis of empowering leadership → resilience at age = Mean ± SD				
Mean − SD (24.26)	0.55	0.09	0.36	0.73
Mean (30.64)	0.72	0.07	0.59	0.85
Mean + SD (36.82)	0.89	0.10	0.69	1.09

*N* = 218. ^**^p < 0.01, ^***^p < 0.001 (two-tailed). Confidence intervals (CIs) are bias-corrected 95% intervals based on 5,000 bootstrap samples. Boot SE, Bootstrap Standard Error; LLCI, Lower Limit of Confidence Interval; ULCI, Upper Limit of Confidence Interval.

To further examine this interaction, a simple slope analysis was conducted (Table 6) by estimating the effect of empowering leadership on resilience at three age levels: the mean, one standard deviation above (+1 SD), and one standard deviation below the mean (−1 SD). For younger employees (−1 SD), the effect was relatively weaker (*b* = 0.55, *p* < 0.001), whereas for older employees (+1 SD), the relationship was substantially stronger (*b* = 0.89, *p* < 0.001). Interpreted in magnitude terms, the slope for older employees is approximately 61.82% steeper than that for younger employees. It indicates that empowering leadership is more strongly associated with resilience among older employees.

**Figure 2:**
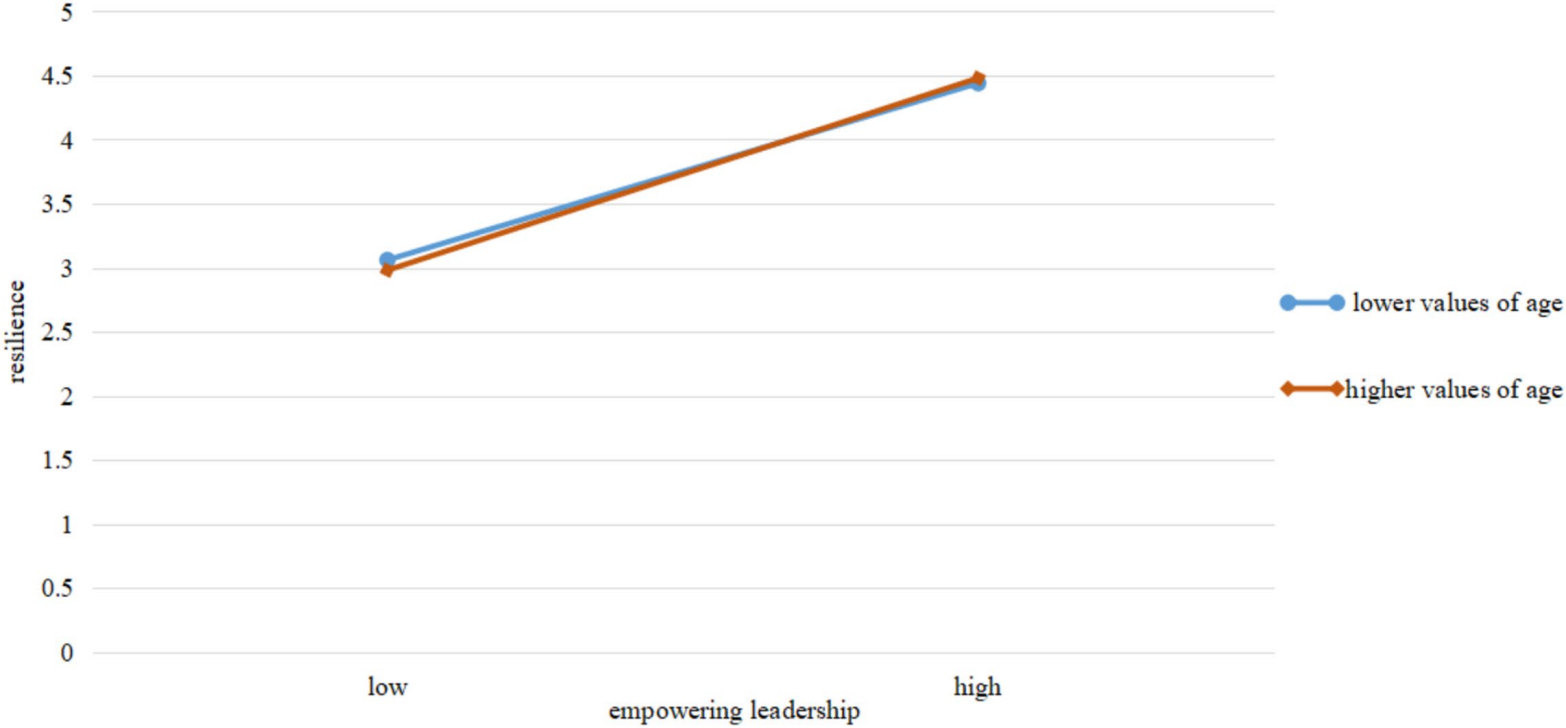
Moderation effect of age.

As illustrated in Figure 2, the simple slope representing the relationship between empowering leadership and resilience is steeper for employees at higher age values (+1 SD) than for those at lower age values (−1 SD). This indicates a stronger positive association between empowering leadership and resilience for older employees, highlighting the practical magnitude of the moderation effect.

Finally, the full moderated mediation model (H6) was tested using the Index of Moderated Mediation, which yielded a significant index (Table 7, Index = 0.01, 95% CI [0.0003, 0.01]). H6 was supported because the CI did not include zero, indicating that the indirect effect of empowering leadership on organizational identification through employee resilience is significantly stronger for older employees. Practically, the index of 0.01 implies that each additional year of age is associated with an increase of 0.01 in the estimated indirect effect, implying a cumulative strengthening of the mediated relationship across the career span.

**Table 7:** Conditional indirect effects and index of moderated mediation.

Age level (moderator)	Indirect effect	Boot SE	Boot LLCI	Boot ULCI
Conditional indirect effects				
Low age (−1 SD)	0.10	0.04	0.04	0.18
Mean age (0)	0.13	0.04	0.06	0.22
High age (+1SD)	0.16	0.05	0.07	0.27
Index of moderated mediation	Index	Boot SE	Boot LLCI	Boot ULCI
EL → Resilience → OI	0.01	0.003	0.0003	0.01

Confidence intervals (CIs) are bias-corrected 95% intervals based on 5,000 bootstrap samples. Boot SE, Bootstrap Standard Error; LLCI, Lower Limit of Confidence Interval; ULCI, Upper Limit of Confidence Interval; EL, empowering leadership; OI, organizational identification.

### 4.4 Robustness check

To ensure the robustness of the hypothesized model and verify that the observed relationships hold across different model configurations, a series of robustness checks were conducted. These checks are critical for validating the findings, as they help assess whether the observed effects are stable and consistent when alternative model specifications are tested. Following recommendations in prior research (Hayes, 2018; Liu et al., 2025), several robustness checks were performed, including the reversal of the mediator and outcome variables and testing alternative moderations.

First, an alternative model was tested where organizational identification was treated as the mediator and employee resilience as the outcome. The robustness check using the reversed model confirmed a significant indirect effect (Effect = 0.23, 95% CI [0.11, 0.37]), providing further evidence of a strong link between resilience and organizational identification. However, this finding does not undermine the proposed causal ordering. Given the time-lagged design, the temporal sequence supports the hypothesized pathway. Thus, the significant reversed effect likely reflects the close conceptual linkage between the two constructs rather than an equivalent causal structure.

Second, to test the stability of the moderated mediation model, the moderator was switched to test if the moderation effect would hold across different paths. Specifically, age was placed as the moderator of the path from employee resilience to organizational identification. The results from this test showed that the moderation effect of age on the resilience → organizational identification path was non-significant (*b* = 0.004, *p* = 0.55), indicating that age does not significantly moderate the relationship between employee resilience and organizational identification.

Overall, these analyses demonstrate that the mediation effect is statistically stable, the temporal design supports the proposed causal sequence, and the moderating role of age is confined to the first stage. Together, these findings strengthen confidence in the robustness and theoretical validity of the model.

## 5 Discussion

This study primarily aimed to examine the psychological mechanisms through which empowering leadership is linked to employees’ organizational identification. The mediating role of employee resilience was identified as the essential psychological bridge in this relationship. Empowering leadership provides a secure base through which resilience is cultivated, and this resilience, in turn, functions as a transformative resource that deepens employees’ sense of oneness with the organization. Importantly, this mediation process was found not to be uniform but to be significantly moderated by employee age. Drawing on the integration of attachment theory and SST, the effectiveness of empowering leadership in fostering resilience and the subsequent indirect effect on organizational identification is more pronounced among older employees. These findings indicate that while resilience represents the universal mechanism through which leadership operates, age serves as a critical boundary condition that determines the strength and efficiency of this developmental pathway.

### 5.1 Transformative power of employee resilience as a mediator

The findings underscore that employee resilience functions as the core mechanism through which leadership behaviors are translated into organizational attitudes. Empowering leadership explained 34% of the variance in resilience. And this is a substantial proportion, given that resilience is traditionally viewed as a stable trait influenced by a complex interplay of personality, life experiences and organizational support (Luthans et al., 2007). The standardized coefficient of *β* = 0.60 for the empowering leadership → resilience path indicates that empowering leadership is strongly associated with employees’ adaptive coping capacity. This association is psychologically meaningful in high-demand contexts where resilience serves as a buffer against strain and facilitates daily coping mechanism in workplaces.

To further clarify the unique contribution of our attachment-based model, it is necessary to situate these results within the broader landscape of resilience research. Traditional studies grounded in SET (e.g., Mao et al., 2022; Li and Zhang, 2022; Caniëls and Hatak, 2022; Çevik and Doğan, 2025) typically conceptualize resilience as a reciprocal response to organizational resources. Under SET, resilience is often viewed as an effort-based obligation. Employees remain resilient to repay the organization’s perceived investment (Mao et al., 2022). In contrast, our findings suggest that resilience is not merely a transactional repayment, but an internalized transformation of the self. When empowering leadership is perceived as a secure-base signal, employees are more likely to restructure their IWMs concerning relational security and competence. This cognitive revision fosters a durable coping capacity that remains resilient, even amidst fluctuations in immediate rewards or instrumental exchanges (Schwarzer, 2024). This shift helps articulate the theoretical novelty of the attachment-based framing relative to a purely transactional logic.

Furthermore, the resilience-based pathway clarifies the unique explanatory power that traditional mediators might overlook. Studies grounded in SET often identify interpersonal trust as the primary mediator connecting leadership to favorable attitudes (e.g., Deluga, 1994; Aryee et al., 2002). Trust is fundamentally a judgement about a leader’s reliability and benevolence (Mayer et al., 1995). It can become more fragile when organizational conditions deteriorate or when resource signals are inconsistent (Mayer et al., 1995). In comparison, resilience reflects an internalized psychological resource that employees carry with them across situations, enabling adaptation even when external support fluctuates (Schwarzer, 2024). In this sense, resilience offers a distinct explanatory pathway for sustaining organizational identification under strain.

Similarly, this study distinguishes resilience from psychological safety, another common mediator of empowering leadership (Lin et al., 2020; Chen et al., 2023). Psychological safety reflects the assurance that one can engage in risky social interactions without enduring punitive outcomes (Kahn, 1990; Edmondson, 1999). While psychological safety describes the quality of the environment, resilience describes a transformative change in the employee’s internal coping capacity and self-regulatory resources (Spinoni et al., 2024). This distinction helps reconcile the “double-edged sword” effect reported in recent literature, where empowering leadership is found to trigger role stress or emotional exhaustion (Cheong et al., 2019). Our results suggest that when empowerment is coupled with relational security of a secure base, it facilitates feelings of mastery and personal growth (Ye et al., 2022) rather than overload. The resulting confidence and gratitude derived from successful adaptation then spill over into stronger collective identity (Lee et al., 2015).

The distinct role of resilience is further evidenced by incremental variance explained. The regression analysis revealed that adding resilience to the model predicting organizational identification increased the explained variance from 50 to 53% (Δ*R*^2^ = 0.03, *p* < 0.01). While this 3% increment may appear modest, it represents the unique variance accounted for by resilience after controlling for the direct effect of empowering leadership and demographic variables. In organizational research, incremental *R*^2^ values of 1–3% for mediation effects are considered practically meaningful (Walters, 2019), particularly when they represent theoretically distinct mechanisms (Sanchez et al., 2024).

To assess model stability, we conducted an additional analysis swapping the mediator and outcome variables. The indirect effect remained significant in this alternative specification (Effect = 0.23, 95% CI [0.11, 0.37]), and was comparable to the hypothesized model (Effect = 0.13, 95% CI [0.06, 0.22]). While this confirms robust interrelations among constructs, it also underscores the need for longitudinal designs to establish causal precedence between resilience and organizational identification.

### 5.2 Employee age as a moderator of the empowering leadership-employee resilience relationship

While prior research has established empowering leadership as a predictor of employee resilience (Kim and Beehr, 2021), the question of for whom this relationship is strongest has remained relatively under-explored. Existing studies have treated empowering leadership as universally beneficial, implicitly assuming that its effects on resilience are uniform across employee populations (Stoverink et al., 2020). The study addresses the theoretical gap by integrating attachment theory with SST to propose and test age as a critical boundary condition. Our findings thus represent theoretical refinement rather than mere empirical replication. We extend the empowering leadership literature by demonstrating that its resilience-building function is age-contingent, with important implications for theory and practice.

Consistent with H5 and 6, the results reveal that the strength of the relationship between empowering leadership and employee resilience is contingent upon employee age, with older employees deriving significantly greater psychological resources from empowering leadership than their younger counterparts. This finding challenges the conventional narrative that empowerment is primarily a youthful aspiration (Maso et al., 2017). It also invites deeper consideration of how the meaning and motivational relevance of empowering leadership evolve across the lifespan (Bal et al., 2016; Bal and Kooij, 2019).

A central debate in life-span development literature concerns whether empowerment is perceived as a growth opportunity or a relational signal (Russo, 2022). Traditional career stage theories suggest that younger employees, who are in the exploration and establishment phases, should be more responsive to empowering behaviors as they seek to build professional competence (Gao, 2023). However, our results align with SST to suggest a different pattern: as the future time horizon shrinks, the psychological value of a secure base outweighs the functional utility of task delegation. For older employees, an empowering leader is perceived not merely as a task facilitator but as a high-value attachment figure whose trust signals a substantial relational investment (Carstensen et al., 1999). This alignment between the socioemotional priorities for older employees and the leader’s secure base function renders the resilience building process more efficient and impactful.

The statistical evidence underscores this disparity. The interaction term explained a significant incremental variance in resilience (Δ*R*^2^ = 0.02, *p* < 0.05), consistent with typical effect sizes for field study moderations (Aguinis et al., 2005). Simple slope analysis revealed that for older employees, the effect of empowering leadership on resilience was *b* = 0.89 (*p* < 0.001), compared to *b* = 0.55 (*p* < 0.001) for younger employees (−1 SD). The slope for older employees was 0.34 units larger, indicating that the positive association between empowering leadership and resilience is more pronounced for older workers. Practically, organizations with aging work forces can expect substantially higher returns on leadership investments. Those targeting younger employees may need to supplement empowerment with additional developmental supports (e.g., structured feedback) to achieve comparable resilience gains.

Furthermore, our findings contribute to the debate on empowerment fatigue among younger generations. Muchtar (2025) suggested that for younger employees, particularly Generation Z, autonomy and empowerment have become normative organizational practices. Young employees treat empowering behaviors from their leaders as standard expectations rather than distinctive motivators (Russo, 2022). Consequently, the resilience-building effect of empowering leadership is notably attenuated among younger cohorts. For these individuals, empowerment is often perceived as a baseline occupational requirement rather than a personalized attachment cue. Conversely, for older employees who may face age-related marginalization or technology-driven obsolescence (Fang et al., 2025), empowerment acts as a powerful restorative resource, reaffirming their relational value within the organization.

Finally, we examined whether this age contingency extends beyond the first stage of the model. An alternative test showed that age did not moderate the resilience → organizational identification path (*b* = 0.004, *p* = 0.55). This confirms that age shapes the interpretation of leadership signals rather than the transformation of psychological resources into organizational identity. By isolating age as a stage-specific boundary condition, this research clarifies that the entire indirect pathway is most potent for older employees. The finding indicates that age not merely functions as a demographic control, but also as a critical lens for understanding the varying efficacy of leadership interventions.

### 5.3 Theoretical contributions

This research makes several significant contributions to the literature on empowering leadership, resilience and lifespan development within organizational contexts. By exploring the psychological mechanisms linking empowering leadership behaviors to employee outcomes, this study offers a more nuanced understanding of how leadership shapes employee connections with their organizations over time, particularly through resilience.

First, this study advances resilience research by identifying employee resilience as the pivotal transformative mechanism linking leadership behaviors to organizational attitudes. Rather than treating employee resilience as a mere end-state of leadership, this study identifies it as the pivotal mediator that links micro-level leadership behaviors to macro-level organizational attitudes. The reasoning behind this shift lies in the capacity of empowering leadership to establish a secure base, which allows resilience to function as a malleable psychological resource. This resource enables employees to reconstruct their relationship with the organization through the successful navigation of adversity. For instance, in high-pressure work environments like the PRD, empowering leadership does more than help employees cope with stress. It provides the psychological infrastructure necessary for them to recover and re-engage, fundamentally strengthening their organizational identification. Ultimately, by clarifying this psychological pathway, the study reframes resilience not as a simple survival trait, but as a functional bridge that converts individualized leader support into a durable organizational bond.

Second, the traditional transactional logic of SET is moved beyond by adopting attachment theory as a novel theoretical lens. Our central premise is that the empowerment process is rooted in emotional-relational foundations rather than the “quid pro quo” resource exchanges often highlighted in existing literature. By conceptualizing the empowering leader as a proximal attachment figure, we argue that resilience is cultivated through relational security and trust instead of simple reciprocity. This theoretical shift allows for a deeper understanding of how employees’ IWMs are shaped and reinforced within the workplace hierarchy. For example, when a leader grants autonomy and expresses confidence, employees interpret these actions not as a contractual deal, but as cues of reliability and availability that stabilize their psychological state during AI-driven displacement anxiety. Consequently, by prioritizing relational attachment over economic exchange, this study provides a more robust and nuanced psychological explanation for how resilience development fosters deep-seated organizational belonging.

Third, employee age is identified as a critical boundary condition that challenges the “one-size-fits-all” approach to empowering leadership. By integrating SST with attachment theory, a lifespan perspective on resilience development is provided. It is demonstrated that the leadership-resilience link is more potent for older employees, indicating that the functional utility of a secure base evolves in accordance with age-related shifts in socioemotional goals. This contribution is particularly significant because it clarifies “for whom” empowering leadership is most effective: older employees, who prioritize emotional meaningfulness and relational investment, are more receptive to the psychological resources offered through empowerment. For this cohort, the autonomy granted by leadership is not merely a task-related affordance but a signal of emotional validation, which reinforces their resilience and strengthens their psychological bond with the organization.

Finally, the relational spillover effect within the moderated mediation framework is further elucidated. Age does not merely moderate a single path but influences the entire indirect process through which leadership affects organizational identity. For older employees, the chain of support is more tightly coupled, from the leader’s secure base to personal resilience and subsequently to organizational identification. This nuanced finding adds demographic precision to the study of resilience, indicating that the bridge from individual capacity to collective identity is constructed differently across various career lifespan stages. In alignment with the SST, the empowering leadership → resilience → organizational identification trajectory is more robust among older employees. This suggests that the relational security offered by leaders is more readily assimilated into their professional identity, resulting in a more enduring organizational connection.

### 5.4 Practical implications

This research offers several implications for organizations operating in the VUCA era, where traditional command-and-control structures are increasingly ineffective in promoting employee resilience and commitment. In the contemporary Chinese workplace, phenomena such as involution (“*neijuan*”) and AI-related job insecurity are becoming increasingly prevalent. These factors significantly heighten anxieties surrounding professional identity and an individual’s enduring relevance in the labor market. Against this backdrop, the following strategies are recommended to help organizations better support their employees and cultivate resilience in this volatile environment.

First, organizations should prioritize the cultivation of employee resilience as a strategic priority in VUCA environments. Given the significant role of resilience in helping employees navigate challenges, this study underscores that empowering leadership is critical for fostering resilience. According to the mediation analysis results, empowering leadership explained 34% of the variance in employee resilience. This indicates that leadership behaviors are not merely peripheral influences but central drivers of resilience. In practical terms, it means that targeted leadership development can yield meaningful improvements in employees’ capacity to navigate adversity. Organizations should therefore treat resilience-building not as an individual responsibility but as a leadership imperative, training managers to function as secure bases who provide psychological safety alongside task delegation.

Practically, organizations should train leaders to delegate meaningful authority, involve employees in decision-making, and create a climate of trust and autonomy. This can be done by ensuring that leadership development programs emphasize the psychological aspect of empowerment, not just functional outcomes. As the study reveals, this shift from transactional leadership to a more relational, empowering leadership style is critical in the modern workplace where uncertainty, AI displacement, and the pressure of involution make resilience essential for survival and engagement.

Second, organizations are encouraged to implement differentiated empowerment strategies for a multi-generational workforce in line with H5 and H6. Specifically, older employees and younger employees perceive and internalize empowerment differently, which can significantly impact their resilience and organizational identification. This suggests that one-size-fits-all leadership strategies may be less effective in the current workplace, especially as generational differences become more pronounced. Our findings also confirm that the empowering leadership → resilience link is significantly stronger for older employees (*b* = 0.72 for the main effect, with age moderating this relationship). This effect size translates to meaningful workplace differences. For older employees, who tend to prioritize emotional security and relational meaning, empowering leadership must be framed in a way that clearly communicates trust and respect. These employees respond strongly to the relational significance of empowerment and signals of trust, such as verbal affirmations or public recognition, can significantly enhance their resilience. In environments shaped by AI displacement anxiety, where older employees may fear irrelevance, these relational cues are crucial for maintaining their psychological engagement and organizational loyalty.

For younger employees, who tend to treat empowerment as a standard organizational expectation, the psychological impact of empowerment must be supported by growth-oriented structures. These employees may not feel the same emotional connection to empowerment unless it is tied to developmental opportunities. Our study shows that younger employees need empowerment combined with tangible developmental support, such as structured feedback and learning opportunities. In practice, this implies that empowering leaders should also create tolerant environments where employees can experiment and learn without fear of reprisal (Zhang et al., 2026). For younger employees, the resilience benefits of empowerment become much stronger when paired with opportunities for skill development and career growth.

Finally, age-aware leadership training and HR practices to ensure that empowerment is consistently effective across different employee groups. Our findings suggest that empowering leadership should not be understood as a uniform practice but should be adapted based on the attachment needs of employees, which change across their careers. By integrating lifespan developmental theories into leadership training programs, organizations can help managers recognize the distinct needs of employees at different stages of life. This is particularly important in light of employee involution and AI-related anxiety, where employees’ emotional and relational needs are crucial to their resilience.

To make this approach practical, organizations should equip managers with tools to diagnose and respond to the attachment needs of their teams. For example, managers could be trained to ask themselves whether an employee needs trust-building cues or growth opportunities to feel supported. HR systems should also track empowerment quality, using surveys to capture employees’ perceptions of relational security and autonomy. These tools will ensure that empowerment is not just about delegating tasks but about aligning leadership behaviors with employees’ developmental and emotional needs.

By implementing age-sensitive leadership and human resource initiatives, organizations can strategically cultivate resilience across diverse generational cohorts. This approach, in turn, bolsters a profound and enduring sense of organizational identification. Our study provides clear guidance on how leadership behaviors should be tailored based on employees’ age and psychological needs, ensuring that all employees, whether older or younger, can thrive in today’s complex and evolving work environment. Given the rise of employee involution and concerns about AI displacement, these strategies offer a way to build long-term engagement, reduce turnover, and promote organizational stability. This can help employees feel both psychologically secure and professionally relevant.

## 6 Limitations and future research directions

Despite the theoretical and practical contributions of this study, several limitations must be acknowledged, highlighting promising directions for future research.

First, although a three-wave time-lagged design was employed to minimize CMB and establish temporal precedence, this study cannot fully support causal inferences. While empowering leadership (T1), resilience (T2), and organizational identification (T3) were measured at different time points to reduce the limitations of the cross-sectional data, the observed relationships remained correlational rather than causal. This concern is further underscored by our robustness check, which revealed that the indirect effect remained significant when the mediator and outcome variables were reversed. This symmetrical finding suggests that resilience and organizational identification may be reciprocally related rather than unidirectional, highlighting the inability of time-lagged design alone to definitively establish causal precedence. Future research could employ longitudinal cross-lagged panel designs or quasi-experimental methods to more rigorously test the causal pathways from leadership behaviors to employee resilience development and subsequent organizational identification.

Second, all variables were assessed using employee self-reports, which may have introduced social desirability or perceptual biases. Although self-reports are appropriate for capturing internal psychological states such as resilience and organizational identification, they are susceptible to biases such as participants presenting themselves in a socially desirable light. Moreover, procedural remedies were employed to minimize CMB, the use of self-report data remains a potential limitation. Future studies should incorporate dyadic or multi-source data (e.g., supervisor assessments of employee resilience or objective performance metrics) to enhance objectivity and provide a more robust validation of the relationship between empowering leadership, resilience and organizational identification.

Third, the geographical and cultural context in which the data were collected may limit the generalizability of the findings. The sample was drawn exclusively from organizations in China’s PRD, a region characterized by high economic dynamism, a unique blend of traditional Chinese values (e.g., respect for hierarchy) and modern global business practices. This unique blend shaped our findings in ways that may not directly translate to other cultural contexts.

Furthermore, the attribution process underlying the link between resilience and organizational identification may differ cross-culturally. In collectivistic cultures, employees are more likely to attribute their adaptive capacity to organizational support, whereas in individualistic contexts, such resilience may be attributed to personal agency, weakening the mediating pathway (Meyer et al., 2012; Hofstede, 2001). Thus, to establish the boundary conditions of the model, future research should pursue cross-cultural comparative designs (e.g., comparing China with low power distance countries such as Denmark) or measure individual-level cultural orientations as more proximal moderators. Such approaches would clarify how culturally-shaped IWMs interact with leadership to shape employee outcomes across diverse settings.

Fourth, Given that cultural socialization strongly shapes age-related IWMs, the observed moderated mediation effect, where older employees respond more strongly to empowering leadership, may differ in Western contexts with lower power distance and stronger individualistic norms (Hofstede, 2001). In such settings, younger employees may expect autonomy as a normative work condition, while older employees, socialized in egalitarian environments, may not interpret empowerment as a distinctive attachment cue (Hofstede, 2001). Consequently, the age-contingent effect was observed could be substantially attenuated.

Age was treated as a proxy for developmental stages of the lifespan, but individual attachment styles were not directly measured. Although the conceptual framework was guided by attachment theory, employees may enter the workplace with pre-existing anxious or avoidant attachment orientations that could interact with leadership behaviors in complex ways. Future research should incorporate assessments of baseline attachment styles to examine how these individual differences combine with age-related socioemotional goals to influence the resilience-building process.

Fifth, the analysis was conducted at the individual level, leaving unexamined potential nested effects. Since empowering leadership often operates at the team level, it can create a climate of collective resilience that influences all members. Future research should employ multilevel modeling to investigate how team-level empowerment interacts with individual-level psychological resources to shape organizational outcomes. Such multi-level extensions would provide a more comprehensive understanding of how secure base dynamics operate across levels of analysis.

Finally, while this study focuses on empowering leadership as a situational activator of secure-based dynamics, employees’ baseline attachment styles (e.g., attachment anxiety or avoidance) were not measured. Attachment theory posits that individuals bring relatively stable IWMs to workplace relationships, which shape how they perceive and respond to leader support (Shaver and Mikulincer, 2007). Employees with secure attachment orientations may be more receptive to empowering leadership and better positioned to internalize it as a resilience-enhancing resource, whereas those with insecure attachment styles might misinterpret or fail to benefit from such leadership. Future research should integrate individual differences in attachment orientations as either moderators of the leadership-resilience link or as control variables to isolate the unique effects of situational leadership.

## 7 Conclusion

Understanding the relational foundations of employee resilience and organizational identification has become a central concern in organizational psychology in response to the intensifying psychological demands of the modern VUCA era. Accordingly, this study developed and empirically tested a moderated mediation model grounded in attachment theory and SST.

First, addressing RQ1, the findings demonstrate that employee resilience serves as the central psychological mechanism through which empowering leadership fosters organizational identification, with empowering leaders functioning as a “secure base” that enables employees to internalize a sense of agency and mastery, thereby strengthening their psychological bond with the organization. This internal transformation, rather than a simple reciprocal exchange, serves as the transformative bridge that strengthens the individual’s psychological bond with the collective identity.

Second, regarding the age-contingent nature of this process (RQ2 and RQ3), the results demonstrate that the effectiveness of empowering leadership is not uniform across the lifespan. Employee age emerges as a critical boundary condition that significantly amplifies the effect of empowering leadership on resilience and the subsequent indirect effect on organizational identification for older employees. For older employees, the secure base provided by empowerment resonates more deeply with their socioemotional priorities, making the resilience-building process more psychologically impactful compared to their younger counterparts. Accordingly, age emerges as a decisive determinant that dictates the ultimate efficiency of empowerment-based management strategies.

This study advances leadership research by shifting from transactional models toward an attachment-based perspective that emphasizes the emotional and relational foundations of resilience. The findings highlight the strategic importance of age-aware management for organizations, particularly those operating in dynamic contexts such as the PRD of China. Rather than adopting a “one-size-fits-all” approach, managers are encouraged to recognize that the functional utility of empowerment varies across the lifespan. By delivering targeted relational support that aligns with the developmental needs of different generations, organizations can foster a resilient, strongly identified, and psychologically sustainable workforce capable of thriving amid uncertainty.

## Data Availability

The raw data supporting the conclusions of this article will be made available by the authors, without undue reservation.
